# Prehabilitation during neoadjuvant therapy in patients with cancer of the upper gastrointestinal tract and rectum—the study protocol

**DOI:** 10.3389/fspor.2024.1495398

**Published:** 2025-02-03

**Authors:** Irina Chmelova, Dalibor Pastucha, Tomas Hudecek, Zdenek Guran, Sona Ciecotkova, Lubomír Martínek, Jana Zubikova, Alena Matlova, Jakub Dolezel, Dana Salounova, Jakub Chmelo

**Affiliations:** ^1^ Department of Rehabilitation and Sports Medicine, University Hospital Ostrava, Ostrava, Czechia; ^2^ Department of Rehabilitation and Sports Medicine, Faculty of Medicine, University of Ostrava, Ostrava, Czechia; ^3^ Department of Surgery, University Hospital Ostrava, Ostrava, Czechia; ^4^ Department of Oncology, University Hospital Ostrava and Faculty of Medicine, University of Ostrava, Ostrava, Czechia; ^5^ Department of Anesthesiology, Resuscitation and Intensive Care Medicine, University Hospital Ostrava, Ostrava, Czechia; ^6^ Centre for Telemedicine Services, University Hospital Ostrava, Ostrava, Czechia; ^7^ Centre for Health Research, Faculty of Medicine, University of Ostrava, Ostrava, Czechia; ^8^ Northern Oesophagogastric Unit, Royal Victoria Infirmary, Newcastle Upon Tyne Hospitals NHS Foundation Trust, Newcastle Upon Tyne, United Kingdom

**Keywords:** prehabilitation, neoadjuvant therapy, physical activity, surgical oncolgoy, physical training

## Abstract

**Aims:**

The concept of prehabilitation, defined as interventions aimed at enhancing a patient's functional capacity prior to an impending physiological stressor, may contribute to reduced postoperative morbidity and mortality. The study's goal is to verify or refute the feasibility of a prehabilitation programme for two diagnostic patient groups during neoadjuvant chemo(radio)therapy, which is prescribed before the scheduled surgical procedure. This is a single group study design, with all patients enrolled receiving the intervention.

**Methods:**

This is an interventional feasibility study of a prehabilitation programme in the form of physical training conducted at home. The training consists of progressively dosed walking and strength exercises for selected muscle groups. Data will be monitored telemetrically and also through telephone contact with participants. Primary outcomes include: the percentage of patients interested in participating in the study out of all patients indicated for neoadjuvant therapy at University Hospital Ostrava during the observed period, the percentage of patients who complete the prehabilitation programme until the date of surgery and individual patient compliance. Secondary outcomes include physical fitness parameters obtained from cardiopulmonary exercise testing, grip strength measured by a dynamometer, changes in body composition, EORTC QLQ-C30 quality of life questionnaire, and a questionnaire on the subjective perception of the exercise programme. Both primary and secondary outcomes will be compared between study arms (two diagnostic groups). The study is registered at ClinicalTrials.gov, ID: NCT05646043.

**Conclusion:**

The results of this study can serve as a foundation for larger, multicentre, controlled studies in the future.

## Introduction

Curative treatment of the cancer of upper gastrointestinal tract (oesophagus, oesophago-gastric junction, and stomach) and treatment of rectal cancer is multimodal. Neoadjuvant chemotherapy, radiotherapy, or chemoradiotherapy (further referred to as neoadjuvant therapy) is commonly indicated during the preoperative period in order to achieve downstaging, downsizing, and to reduce the risk of recurrence.

Oncological treatment has a negative impact on the overall physical and mental condition of patients; their quality of life and daily activities are reduced (1). Neoadjuvant therapy is associated with a reduction in cardiopulmonary function among patients undergoing oesophagectomy (2). The decline in cardiorespiratory reserve following neoadjuvant chemotherapy in individuals with oesophagogastric cancer is observed to persist up to the time of surgery (3, 4). The surgical procedures performed are both demanding and prolonged, imposing a significant burden on patients. Furthermore, the duration of surgery correlates with a statistically significant increased risk of complications, including wound infection, dehiscence, hemorrhage, and pneumonia (5).

In the traditional care model, most patients receive rehabilitation in the postoperative period. The rehabilitation aims to improve overall physical fitness, regain self-care abilities, and return to activities patients were able to do before the oncological diagnosis, including returning to their profession and hobbies. One of the main aim of early rehabilitation in postoperative time is to improve the mobility of the patient. Mobilization soon after surgery has a positive effect on recovery of function and therefore it is a part of ERAS (Enhanced Recovery After Surgery) programmes (6). ERAS is becoming a standard part of clinical practice, positively contribute to reducing the risk of postoperative complications, and shortening the length of stay (7–9). Despite the positive effect of ERAS measures, especially the surgery of the upper gastrointestinal tract, is still associated with a high morbidity and mortality. Cardiopulmonary fitness and muscle strength are reduced in these patients; they suffer from fatigue, anxiety, depression, and have lower quality of life (1, 10–14). These accompanying problems negatively influence the therapeutic process, and therefore, lately, attention and efforts to improve postoperative outcomes are increasingly focusing on the period before surgery.

The concept of prehabilitation, which is defined as an intervention improving an individual's functional capacity before an upcoming physiological stressor (15, 16), might be a way to improve outcomes. The prehabilitation is commonly multimodal, with the main components being physical exercise, nutritional interventions and psychological coaching (17). Despite the inconclusive evidence regarding the efficacy of prehabilitation, programmes are increasingly being integrated into clinical practice. A randomized controlled trial published in 2022 demonstrated that patients undergoing prehabilitation prior to oesophagogastric cancer resection exhibited greater retention of cardiopulmonary fitness, muscle mass, and quality of life compared to those receiving standard care (18).

Furthermore, several studies indicate a significantly lower incidence of complications among the prehabilitated cohort, alongside reduced hospital stays and enhanced functional capacity (19–24). An umbrella review of systematic reviews concerning prehabilitation for surgical patients highlights low to very low certainty evidence supporting a reduction in complications and length of stay in cancer surgery. Additionally, moderate certainty evidence indicates improvements in functional recovery, while low to very low certainty evidence suggests that nutritional prehabilitation may mitigate the risks of complications, mortality, and length of stay (25).

The positive influence of physical activity and exercise is undeniable. Physical activity reduces the risk of cardiovascular diseases (26), type II diabetes (27), and cancer (28). On the contrary, physical inactivity is directly related to mortality (29). Physical activity prior to cancer diagnosis (30), but also after oncological treatment affects survival (31) and might be associated with disease recurrence (32). Regular exercise leads to the body's adaptation to physical stress and increases fitness. Although scientific interest in prehabilitation is on the rise, there are many ambiguities and questions that must be answered. The 15-week programme for patients prior to oesophagogastric cancer resection, consisting of aerobic, resistance, and flexibility training, included twice-weekly supervised one-hour exercise sessions combined with thrice-weekly one-hour home exercises and a nutritional and psychological intervention. This approach has been shown to improve cardiopulmonary fitness, muscle strength, and quality of life (QOL) as previously mentioned (18). Ten week supervised training programme (aerobic exercise, muscle strength and endurance) et dose 150 min per week may enhance physical function during neoadjuvant treatment in rectal cancer patients (33). There is not enough scientific evidence whether programmes carried out under direct expert supervision are more effective than those done by the patient independently at home.

These questions should be answered before prehabilitation programmes become a standard part of clinical practice. Not only for this reason, authors decided to conduct the study presented here.

## Methods

This is a feasibility study focused on patients with oesophagogastric cancer or rectal cancer who were, based on the decision of the multidisciplinary team (MDT) of the University Hospital in Ostrava (FNO), indicated for neoadjuvant therapy in the preoperative period.

These groups of patients were chosen as they are commonly treated with neoadjuvant treatment, which opens a time window for the application of a prehabilitation programme. The prehabilitation programme is designed in the form of physical training aimed at improving or retaining cardiorespiratory fitness as well as the strength of selected muscle groups. Each patient also receives nutritional support. The programme is designed to minimise significant time demands on medical staff, so it will be implemented in the patient's home environment. The study aims to answer question whether the selected patients will be able to complete a home exercise regimen during the preoperative period when they undergo neoadjuvant therapy.

The primary outcomes to evaluate the feasibility of the study are:

1. Percentage of patients who consent to participate in the study out of all diagnosed and for neoadjuvant therapy indicated patients in the monitored period. The reasons for refusing to participate will be recorded.
2. The percentage of patients who consent to participate in the study and who remain participants until the end of the study period, out of all consented patients.
3. Individual patient compliance—defined as the number of days the patient wore a smart bracelet, carried out or attempted to complete the prescribed exercise programme (aerobic exercise and strength training), and remained accessible for follow-up telephone contact, out of the total number of days in the observed period.
4. All adverse events related to the prehabilitation programme implementation.

Secondary outcomes include changes in physical fitness parameters obtained from cardiopulmonary exercise testing CPET—peak oxygen uptake, anaerobic threshold test performed on a Bicycle ergometer ERGOSELECT 5 (Ergoline GmbH, Bitz, Germany and COSMED srl, Rome, Italy) using a ramp protocol with a gradual increase in load of 0.5 W per kilo per min, changes in grip strength measured by hand dynamometer, changes in body composition, Borg's scale of perceived exertion, changes in health-related quality of life measured by EORTC QLQ-C30 quality of life questionnaire, and a subjective perception of the exercise programme.

Inclusion criteria for this study are age >18 years and planned resection for locally advanced oesophagogastric adenocarcinoma or adenocarcinoma of rectum with planned neoadjuvant therapy and ability to complete CPET and able to sign informed consent for study participation. Exclusion criteria for this study are contraindications to CPET or inability to perform CPET or daily exercises due to orthopaedic limitations (e.g., amputation, severe gonarthrosis, coxarthrosis) or inoperability determined by the MDT decision or synchronous malignant disease or multivisceral resection or planned non-surgical therapeutic procedures.

The sample size was set at 40 patients given the feasibility nature of the study (34). The patient will be referred to oncologist once MDT agrees on the therapeutic plan. The oncologist, overseeing the oncological treatment, will inform the patient about the possibility of participating in the study and if patient agrees to participate in the study, informed consent will be signed before start of the neoadjuvant therapy.

**Figure 1:**
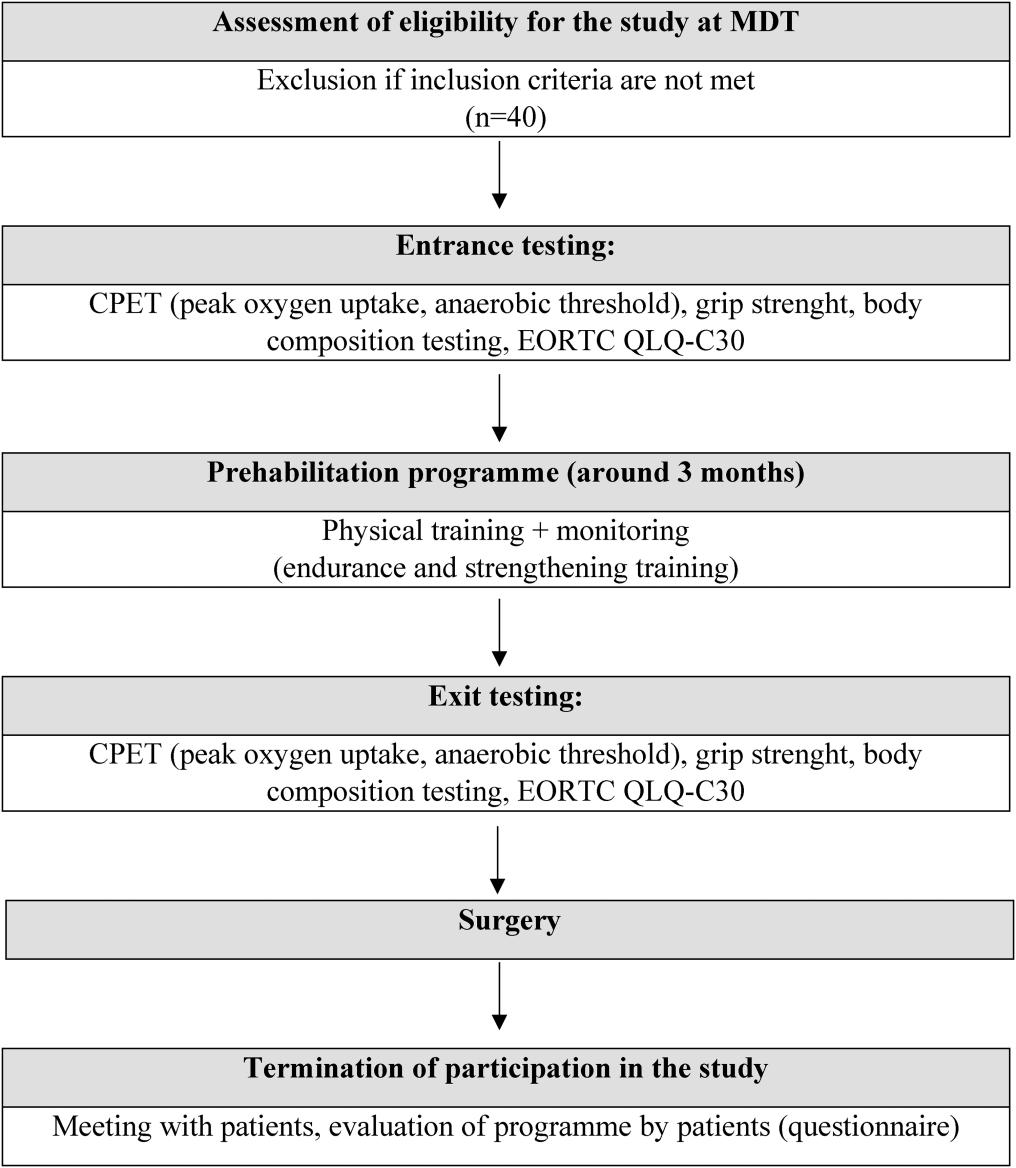
Study diagram (MDT, multidisciplinary team; CPET, cardiopulmonary exercise testing).

The length of the prehabilitation programme is based on the length of oncological treatment which for group of patients with oesophagogastric adenocarcinoma consists of four cycles of neoadjuvant chemotherapy at 15-day intervals for a total length of 60 days. The restaging time takes 14–21 days, and time from restaging to surgery usually takes 7–14 days. The total duration for the prehabilitation programme for this group of patients will be about 11 to 13 weeks. For rectal adenocarcinoma, the oncological treatment involves 4–5 weeks of chemo(radio)therapy, followed by restaging after a 3–4 week interval and followed by surgery 1–2 weeks later. The total duration of the programme for this diagnostic group will be around 8 to 11 weeks (Figure 1).

## Intervention

### Prehabilitation programme

In addition to psychological and nutritional support, which is a standard part of cancer treatment, the foundation of the prehabilitation programme is physical training. The nutritional support of probands enrolled in the study is similar to that of other cancer patients. Every patient is screened and assessed by a dietitian for potential nutritional supplementation as part of the standard care. This is followed by tailored dietetic advice regarding food, protein, energy intake, and supplementation. In some cases, patients may require enteral or parenteral feeding, which is occasionally administered through adjuncts such as nasoenteric tubes, feeding jejunostomies, or, in rare instances, total parenteral nutrition.

Even before starting neoadjuvant therapy, during the first clinical examination, the patient will be thoroughly educated about the prehabilitation programme, and they will be given an exercise diary to record their daily physical activity.

The initial examination will include an assessment of the overall condition, including obtaining past medical history and testing grip strength. Validated InBody230 body composition analyser (Biospace Co., LTD., Coalville, United Kingdom), which fulfils the standards of IEC60601-1(EN60601-1), will be used to analyse body composition (percentage of water, fat, muscle mass). Patients will be educated on the use of technologies that will record monitored parameters (steps, heart rate, weight), and they will be given a telemetry set—a MiBand 5 pedometer (Xiaomi Inc, Beijing, China), a Galaxy A13 mobile phone (Samsung Electronics, Suwon, South Korea), a W62 telemetry scale (Zhongshan Loease Electronic Technology Co, Ltd, Zhongshan, China), and an initial CPET will be performed.

For one week, the patient's physical activity will be monitored telemetrically by recording the number of steps. Based on this, a baseline value will be established, from which the subsequent exercise dosage will be derived.

### Physical training

Physical training will consist of endurance (walking) and strength components. Walking is the most natural physical activity for humans. The training programme aims to be individualised by the research team. The basis for determining the number of steps per day is the baseline value for each patient. This will be increased by 10%–15% every week until the date of surgery. If the increase is not tolerated by the patient, the last increased step count will be retained, and consideration of further increase will be postponed until the following week. The expected total increase in steps is 120%–180% of the baseline. The authors are aware that not every patient can achieve these values. Within the feasibility study, the authors want to verify the acceptable dosing of the load. At the same time, authors' goal is for the patient to perceive their load at medium intensity (measured by the Borg scale, corresponding to 3–4 out of 10) for 30 min per day.

Strength training will be applied using exercises to strengthen the upper (biceps and triceps groups through exercises without and with dumbbels or rubber expander, wall push ups) and lower limbs (strengthening of gluteal, quadriceps, hamstrings and culf muscles through squats with or without back support, standing up from a chair, toe raises, leg raises) and the core. Training will involve a set of eight exercises in several difficulty variations. The patient chooses a variant that he or she can perform and gradually can choose a more difficult variant of the exercise. Each exercise will be done for one minute. The set will be repeated twice. The total strength training time will take approximately 20 min. The patient will perform this set seven days a week. Achievement of Borg 3–4 for strength training is not required. A description of each exercise and photo documentation is part of the exercise diary. The patient is also taught about possible mistakes when performing each exercise and the mistakes are photo documented. The education in the training program is part of the first clinical examination.

If interested, the patient can undertake additional activities (running, cycling, etc.) during the study. Patients will be encouraged to do these activities, and these will be recorded.

Patients will record their daily physical activity in their exercise diaries (number of steps per day, strength training, subjective perception of intensity according to Borg, other physical activities).

Physical training will be carried out throughout the neoadjuvant therapy and will continue after its completion, up to 1–2 weeks before surgery.

### Data monitoring

Data collection will be obtained in two ways: by contacting patients by phone and by remote monitoring. During physical training, the patient will be contacted by phone once a week by the study researcher to support them, monitor data from the exercise diary (number of steps per day/week, number of days per week performing strength exercises, subjective perception of intensity according to the Borg scale during aerobic activites, other physical activities from the previous week). During the conversation, any patient difficulties and problems will be discussed. The patient will have the opportunity to participate in specifying the physical load (number of steps) for the following week.

Remote monitoring will be ensured in collaboration with the telemedicine services centre. Patients will be equipped with the aforementioned telemetry set to ensure data capture. Data will be transmitted continuously to the research team and evaluated once a week before a phone call with the patient.

The telemedicine service centre also includes a control centre, which will monitor threshold and critical values, track trends of measured values, identify and display incidents (alerts and alarms), generate reports, secure store and archive data, transfer data from client terminals to the monitoring centre, and provide technical support to system users.

### Termination of the prehabilitation programme

The exercise programme will be terminated 1–2 weeks before the scheduled surgical procedure. During this period, a control CPET will be carried out, a control examination of the grip strength of the dominant hand, a control questionnaire of the quality of life, and the patient's weight will be measured. During the postoperative period (within 1–2 weeks after surgery depending on his current state of health) a questionnaire survey of the patient's subjective perception of the exercise programme will be conducted.

## Statistical methods

The collected data will be entered into electronic forms. Basic descriptions of individual groups will be made using descriptive statistical methods. Continuous normally distributed variables will be presented as mean and standard deviation, skewed continuous variables and ordinal variables as median and interquartile range. Categorical data will be presented as numbers and percentages. Demographic characteristics will be compared between groups using a *t*-test or a Mann-Whitney U test (age) and a chi-square or Fisher's exact test (gender). Normality of data will be assessed by a Shapiro-Wilk test. Primary outcomes will be reported as numbers and percentages. To determine the significance of changes during the monitored period, for metric data (peak oxygen uptake, anaerobic threshold, number of steps, grip strength), depending on their distribution, a paired *t*-test or a paired Wilcoxon test will be used. The paired Wilcoxon test will also be used for ordinal data (scores from questionnaires). Comparisons between two diagnostic patient groups for continuous data, again depending on the properties of their distribution, will be performed using a two-sample *t*-test or a Mann-Whitney U test, and for ordinal data, a Mann-Whitney U test. *P*-values less than 0.05 will be considered statistically significant. Statistical analyses will be performed using the IBM® SPSS® Statistics version 28 (IBM, Armonk, USA).

## Conclusion

The aim of the study is to demonstrate or refute the feasibility of the prehabilitation programme in the form of physical training for two diagnostic patient groups (oesophagogastric adenocarcinoma and rectal adenocarcinoma) during neoadjuvant chemo(radio)therapy, which is indicated before the planned surgical procedure. The study aims to answer the basic research question of whether, during the preoperative period of neoadjuvant therapy, selected patients will be able to complete a home-based exercise regimen.

The study's investigators anticipate that the set prehabilitation exercise programme for patients undergoing neoadjuvant chemo(radio)therapy will be safe and feasible. The results of the study may form the basis for larger, multicentric, controlled studies in the future.

The main limitation of this study is the necessity for coordination between a diverse interdisciplinary team of specialists (oncology, surgical rehabilitation, functional diagnostics, telemetry, nutrition, etc.). Due to the high workload of these specialised outpatient clinics, this may result in a prolonged period before the proband is included in the prehabilitation programme. The extensive range of clinical conditions and physical fitness levels of the probands prior to their inclusion in the study may be the underlying reason for the inability to initiate prehabilitation. Moreover, the rapid advancement of technology in the field of telemedicine may introduce novel forms and applications of telemetry throughout the course of the study.
